# Longitudinal Changes in Total Brain Volume in Schizophrenia: Relation to Symptom Severity, Cognition and Antipsychotic Medication

**DOI:** 10.1371/journal.pone.0101689

**Published:** 2014-07-18

**Authors:** Juha Veijola, Joyce Y. Guo, Jani S. Moilanen, Erika Jääskeläinen, Jouko Miettunen, Merja Kyllönen, Marianne Haapea, Sanna Huhtaniska, Antti Alaräisänen, Pirjo Mäki, Vesa Kiviniemi, Juha Nikkinen, Tuomo Starck, Jukka J. Remes, Päivikki Tanskanen, Osmo Tervonen, Alle-Meije Wink, Angie Kehagia, John Suckling, Hiroyuki Kobayashi, Jennifer H. Barnett, Anna Barnes, Hannu J. Koponen, Peter B. Jones, Matti Isohanni, Graham K. Murray

**Affiliations:** 1 Department of Psychiatry, Institute of Clinical Medicine, University of Oulu, Oulu, Finland; 2 Department of Psychiatry, Oulu University Hospital, Oulu, Finland; 3 Department of Psychiatry, University of Cambridge, Cambridge Biomedical Campus, Cambridge, United Kingdom; 4 Behavioural and Clinical Neuroscience Institute, University of Cambridge, Cambridge, United Kingdom; 5 Institute of Health Sciences, University of Oulu, Oulu, Finland; 6 Unit of General Practice, Oulu University Hospital, Oulu, Finland; 7 Department of Diagnostic Radiology, Oulu University Hospital, Oulu, Finland; 8 VU University Medical Centre, Department of Radiology, Amsterdam, The Netherlands; 9 Department of Neuroimaging, Institute of Psychiatry, King's College London, London, United Kingdom; 10 Department of Neuropsychiatry, School of Medicine, Toho University, Tokyo, Japan; 11 Cambridge Cognition Ltd, Bottisham, Cambridge, United Kingdom; 12 Institute of Nuclear Medicine, University College London Hospitals NHS Foundation Trust, London, United Kingdom; 13 University of Eastern Finland, Faculty of Health Sciences, Institute of Clinical Medicine and Department of Psychiatry, Kuopio University Hospital, Kuopio, Finland; United (Osaka U, Kanazawa U, Hamamatsu U Sch Med, Chiba U and Fukui U) Graduate School of Child Development, Japan

## Abstract

Studies show evidence of longitudinal brain volume decreases in schizophrenia. We studied brain volume changes and their relation to symptom severity, level of function, cognition, and antipsychotic medication in participants with schizophrenia and control participants from a general population based birth cohort sample in a relatively long follow-up period of almost a decade. All members of the Northern Finland Birth Cohort 1966 with any psychotic disorder and a random sample not having psychosis were invited for a MRI brain scan, and clinical and cognitive assessment during 1999–2001 at the age of 33–35 years. A follow-up was conducted 9 years later during 2008–2010. Brain scans at both time points were obtained from 33 participants with schizophrenia and 71 control participants. Regression models were used to examine whether brain volume changes predicted clinical and cognitive changes over time, and whether antipsychotic medication predicted brain volume changes. The mean annual whole brain volume reduction was 0.69% in schizophrenia, and 0.49% in controls (p = 0.003, adjusted for gender, educational level, alcohol use and weight gain). The brain volume reduction in schizophrenia patients was found especially in the temporal lobe and periventricular area. Symptom severity, functioning level, and decline in cognition were not associated with brain volume reduction in schizophrenia. The amount of antipsychotic medication (dose years of equivalent to 100 mg daily chlorpromazine) over the follow-up period predicted brain volume loss (p = 0.003 adjusted for symptom level, alcohol use and weight gain). In this population based sample, brain volume reduction continues in schizophrenia patients after the onset of illness, and antipsychotic medications may contribute to these reductions.

## Introduction

Over a century after Emil Kraepelin [1] used the term dementia praecox (early dementia) to describe schizophrenia, it remains debatable whether there are progressive neuroanatomical changes in schizophrenia. According to two meta-analyses, there is, on average, greater loss of brain volume over time in schizophrenia compared to controls [2], [3]. However, the cause for progressive brain tissue loss in schizophrenia remains unknown. It is possible that the disease process itself is causal [4] but it is also critical to address the potential contributions of iatrogenic factors such as antipsychotic medication [5].

It is also crucial to examine the significance of brain volume change in schizophrenia in terms of impact, if any, to the patient. Some studies have suggested that patients with the worst clinical outcomes may also exhibit the most extensive brain atrophy over time [6]–[8]. Cross-sectional and short/medium-term (under three years) studies associate cognitive impairment with smaller brain volumes [2], consistent with the results of a long-term study which also showed that progressive brain volume decrease is associated with poor cross-sectional cognition [9].

Neuroimaging findings in studies of schizophrenia are particularly sensitive to patient selection and inappropriate control group comparisons, highlighting these factors as significant sources of bias [10]–[12]. Longitudinal studies of brain structural change must therefore examine schizophrenia and control samples that represent as accurately as possible the majority of the patient and general populations from which they are drawn. In contrast to the majority of studies on clinic or hospital samples [12], population-based studies meet this criterion, so that any findings on brain structure and prognosis in schizophrenia have more wideranging implications.

## Aims and hypotheses

We had the opportunity to study brain changes in schizophrenia and control participants in an epidemiologically principled sample. The cases were not drawn from psychiatric services, but from a large general population-based birth cohort. The control participants were randomly drawn from the same birth cohort, and had no history of psychotic episode. Due to the setting, both groups were matched for age (year of birth) and place of birth.

We tested the following main hypotheses:

1. Participants with schizophrenia would show a greater decrease in total brain volume than control participants.
2. Within the schizophrenia group, the decrease in total brain volume would be associated with deterioration in symptom severity, level of function, and cognition.
3. Within the schizophrenia group, the degree of exposure to antipsychotic medication would be associated with decrease in total brain volume.

In additional analyses, we explored the regional basis of group differences in brain volume change and of associations between antipsychotic medication exposure and brain volume change.

## Methods

### Ethics statement

Permission to gather data was obtained from the Ministry of Social and Health affairs, and the study design has been approved by the Ethical Committee of the Northern Ostrobothnia Hospital District, Oulu, Finland. Written informed consent was obtained from all participants. In a case of possible compromised capacity to consent, written informed consent was obtained also from a close relative of the participant.

The study was conducted according to the principles expressed in the Declaration of Helsinki.

### Setting

We compared whole brain volume change over time in participants with schizophrenia and control participants in a general population birth cohort. In participants with schizophrenia we explored the whole brain volume change in relation to symptom severity, level of function, cognition, and use of antipsychotic medication.

### Participants

The participants of the present study are members of the Northern Finland Birth Cohort 1966 (NFBC1966; www.oulu.fi/nfbc). The NFBC1966 is an unselected population birth cohort ascertained during mid-pregnancy. The birth cohort is based upon 12,068 pregnant women and their 12,058 children with an expected delivery date during 1966 [13]. The present study is based on 10,934 individuals living in Finland at age 16 who allow the continued use of their data. Data from the NFBC1966 is publicly available. Researchers wishing to utilise the data should apply through the procedures described at www.oulu.fi/nfbc.

The Finnish Hospital Discharge Register (FHDR) was used to identify NFBC 1966 members with psychosis. The FHDR covers all mental and general hospitals, and beds in local health centres nationwide. In the FHDR, psychiatric diagnoses were coded routinely between 1969 and 1986 using ICD-8, between 1987 and 1995 using ICD-9, and since 1996 using ICD-10. Until recent years, the majority of patients experiencing an episode of schizophrenic psychosis in Finland were hospitalized [14]. All cohort members over the age of 16 years appearing on the FHDR until the end of 1997 for any mental disorder (ICD-8 290-309, ICD-9 290-316, and ICD-10 F00- F69, F99) were identified. All case records were scrutinized and diagnoses were validated using Diagnostic and Statistical Manual of Mental Disorders Third Edition Revised (DSM-III-R) criteria [15], [16].

**Figure 1:**
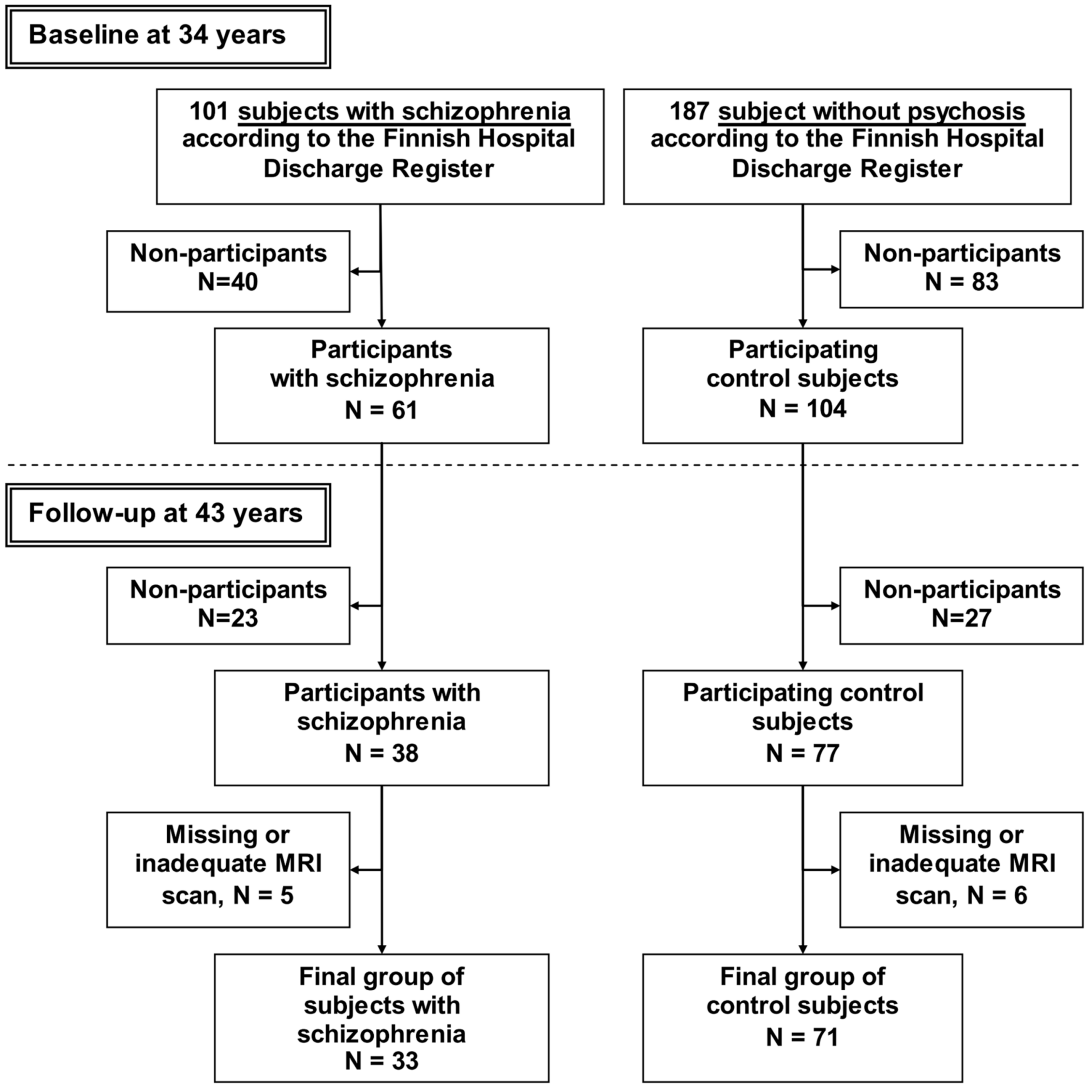
Flow-chart of the study.

A total of 146 cohort members with history of psychosis (including 101 with a diagnosis of schizophrenia) were invited to participate by letter, including three cases with only outpatient treatments and not recorded in the FHDR (Figure 1). The baseline MRI study was conducted in 1999–2001 when participants were aged 33–35 years. Structured Diagnostic Interview for DSM-III-R [17] (SCID) and anamnestic information including individual hospital case notes were the main diagnostic instruments. Of the 92 participating psychosis participants, 61 were diagnosed as having schizophrenia. Participants with schizophrenia had an average illness onset 11 years before the baseline MRI scan [18].

The control participants were randomly selected from the NFBC 1966 members living in the Oulu area; they had not had a psychotic episode according to the FHDR by 1997. Altogether, 187 comparison cohort members were invited and 104 (56%) participated. After a complete description of the study, written informed consent was obtained from all participants [11].

All participants who had undergone a baseline MRI scan were invited to a follow-up examination including a repeat MRI scan, after a nine year interval (during 2008–2010). Thirty eight (61%) of the participants with schizophrenia and 77 (74%) of the control participants participated in the follow-up. (One of the control participants had had a psychotic episode during the follow-up period according to the FHDR and was not included in the final study group.) SCID-IV [19] was used as a diagnostic instrument, supplemented by anamnestic information (including hospital case notes until the year 2009). The original diagnosis was upheld at follow up for all participants with schizophrenia on the basis of the SCID-IV and case note review.

For five participants with schizophrenia and six controls MRI datasets were incomplete (scans missing at either time-point). As a result, the final schizophrenia group included 33 participants and the control group 71 participants (Figure 1). Life-time DSM-III-R diagnoses for schizophrenia included eight participants with disorganized schizophrenia, six with paranoid schizophrenia and 19 with undifferentiated schizophrenia.

### Background information of the participants

Nineteen (58%) of the participants with schizophrenia were male and 14 (42%) female; and 43 (61%) of the control participants were male (Table 1). The mean age at baseline was 33.7 years in participants with schizophrenia and 34.6 years in control participants. At follow-up the respective figures were 42.8 years in participants with schizophrenia and 43.0 years in control participants. The mean follow-up time was 9.1 years (SD 0.6, range 7.5–10.2 years) for schizophrenia participants and 8.5 years (SD 0.7, range 7.0–10.4 years) for the control participants.

**Table 1:** Sociodemographic background and handedness in the schizophrenia and control groups.

Variable	Schizophrenia	Controls
	N = 33	N = 71
**Gender**		
Male N (%)	19 (58 %)	43 (61%)
Female N (%)	14 (42%)	28 (39%)
**Age at first time point**		
Mean age, years (SD)	34 (0.6)	35 (0.7)
Range, years	33–35	33–36
**Age at second time point**		
Mean age, years (SD)	43 (0.5)	43 (0.5)
Range, years	42–44	42–44
**Educational level**		
Basic (<9 years) N (%)	7 (21%)	3 (4%)
Secondary (9–12 years) N (%)	26 (79%)	45 (63%)
Tertiary (>12 years) N (%)	0 (0%)	23 (32%)
**Maternal educational level**		
Basic (<9 years) N (%)	14 (42%)	50 (70%)
Secondary (9–12 years) N (%)	14 (42%)	20 (28%)
Tertiary (>12 years) N (%)	2 (6%)	1 (1%)
Missing N (%)	3 (9%)	-
**Marital status at first time point**		
Married or cohabiting N (%)	9 (27%)	54 (76%)
Single N (%)	23 (70%)	17 (24%)
Missing N (%)	1 (3%)	-
**Handedness**		
Right N (%)	30 (91%)	65 (92%)
Left N (%)	2 (6%)	5 (7%)
Both N (%)	0 (0%)	1 (1%)
Missing N (%)	1 (3%)	-

Compared to the control group, the schizophrenia group had a lower level of educational attainment and were more likely to be single (unmarried, divorced, widowed and not cohabiting) rather than married or cohabiting. In both groups, more than 90% were right-handed.

### Alcohol use

Alcohol use was assessed using same questionnaire [20] in the baseline and follow-up assessments. The frequency and amount used was converted to mean grams of alcohol used per day. Mean daily alcohol use across both assessments was log transformed (given the skewness of the distribution). There was no significant difference in alcohol use between groups (independent samples t-test p = 0.78) (schizophrenia group: mean 12.7 grams, SD 34.7, median 2.0; control group: mean 11.0 grams, SD 10.6, median 6.7)

### Use of recreational drugs

Urine samples at both assessments were obtained from all participants apart from one schizophrenia participant and one control participant at the baseline assessment. Urinalysis revealed that three schizophrenia patients and one control participant had used opioids at either assessment. None of the participants had used cannabis, cocaine or amphetamine.

### Weight change

At follow up, participants with schizophrenia had gained significantly more weight compared to control participants (independent samples t-test p<0.001) (schizophrenia group: mean 9.4 kg, SD 10.5; control group: mean 2.0 kg, SD 6.1).

Weight change and alcohol use were used as covariates in schizophrenia versus control participants analyses as they have been connected to brain volume reduction [21], [22].

### Clinical characteristics of participants with schizophrenia

Age of onset was defined according to the first hospitalization due to a psychotic episode. The mean onset age was 22.5 years (median 21.3 years) (Table 2). The mean time from illness onset to the baseline scan was 11.1 years and 20.2 years to the follow-up scan.

**Table 2:** Clinical characteristics of participants with schizophrenia.

Variable	Mean (SD)	Range
**Age of onset (years)**	22.5 (4.3)	16.7–31.0
**Duration of illness (years) at baseline ataselineyears)**	11.1 (4.2)	2.8–18.6
**Duration of illness (years) at 9 year follow-up**	20.2 (4.4)	11.9–26.8
**Total PANSS** a **score at baseline**	56.4 (20.2)	30–102
**Total PANSS** a **score at 9 year follow-up**	76.7 (26.4)	39–130
**Mean total PANSS** a **score**	67.3 (22.1)	34.5–116
Mean negative symptom subscore	18.6 (8.6)	6.5–33.5
Mean positive symptom subscore	19.7 (6.0)	10–30
Mean cognitive symptom subscore	24.4 (8.7)	10–40.5
Mean excitement symptom subscore	14.2 (3.8)	8.5–27.5
Mean depression symptom subscore	16.6 (4.7)	9.5–27.5
**SOFAS** b **score at baseline**	45.3 (17.1)	3–90
**SOFAS** b **score at 9 year follow-up**	48.5 (17.0)	24–82
**Mean SOFAS** b **score**	46.9 (15.7)	13.5–86
**Typical antipsychotics (dose years** c **)**	8.2 (12.2)	0–48.2
**Atypical antipsychotics (dose years** c **)**	22.8 (20.7)	0–65.1
**Any antipsychotics (dose years** c **)**	31.0 (26.9)	0–104.8

a PANSS, Positive and Negative Syndrome Scale, assessment of the PANSS in the baseline and 9 year follow-up are not completely comparable (please see text in methods).

b Social and Occupational Functioning Assessment Scale.

c Equivalent to 100 mg daily chlorpromazine during the 9-year follow-up.

The Positive and Negative Syndrome Scale, PANSS [23] was administered at each assessment. We analysed the total PANSS score and scores of five subscales: negative, positive, cognitive, excitement and emotional [24]. A mean score of 67 in the total PANSS reflects ‘moderately ill’ patients [25]. The mean total PANSS and subscales scores at each assessment, as well as the change score from the first to the second assessment, were analysed. The procedure for measuring the PANSS differed at the two time-points; on the day of the first scan it was measured after a general psychiatric interview and the SCID, whilst on the day of the second scan, a PANSS specific interview was used.

The Social and Occupational Functioning Scale, SOFAS (DSM-III-R version) was used at both assessments to rate social and occupational functioning during the previous week [26]. The mean SOFAS score of the two assessments and change score were used in the analysis. Higher scores reflect better functioning. The mean score of 47 in schizophrenia participants across the assessments reflects ‘serious impairment in social, occupational, or school functioning’. One subject with history of schizophrenia scored 90 in the SOFAS at baseline and 82 at follow-up, and was estimated as fully recovered [27], but was not excluded from the schizophrenia group.

### Antipsychotic medication during the follow-up period

Current and earlier use of antipsychotic medication was ascertained by interview for all participants at both assessments. Additionally individual hospital and outpatient case notes were carefully reviewed to assess the use of antipsychotic medication during the follow-up. During the follow-up three schizophrenia participants took no antipsychotic medication and two took antipsychotic medication for less than one dose year. We measured use of antipsychotic medication in terms of chlorpromazine equivalents [28], and we expressed the total use of antipsychotic medication over the study period in terms of the number of dose years of 100 mg of chlorpromazine daily.

Dose years for any antipsychotic was used as a continuous variable in the primary analysis and for typical and atypical antipsychotics separately in secondary analyses. A log transform was applied given the skewness of the distribution and the natural logarithm of antipsychotic dose-years exposure was used as the predictor variable in our analyses.

### Brain imaging: data acquisition

The participants were scanned at baseline and follow up with the same 1.5 T GE Signa (General Electric, Milwaukee, Wisconsin) scanner bore in Oulu University Hospital. The scanner was used to obtain T1-weighted high-resolution three dimensional spoiled gradient echo (3D SPGR) images at baseline. The images were acquired in the coronal plane covering the whole brain (slice thickness 1.5 mm; in-plane resolution matrix size 256×256; voxel size 1.5 mm×1 mm×1 mm; repetition time 35 ms; echo time 5 ms; flip angle = 35). Prior to the second time point the scanner was up-graded into HDxt with a new gradient system and parallel image data acquisition with an 8 channel receiving coil. At follow-up, the T1 weighted images were acquired, with a 3D fast spoiled gradient echo (FSPGR) sequence (slice thickness  = 1 mm; in-plane resolution matrix size 256×256; voxel size 1 mm×1 mm×1 mm; repetition time 12.576 ms; echo time 5.3 ms; and flip angle  = 20).

### Brain imaging: data processing

For neuroimage processing, we used the SIENA (Structural Image Evaluation, using Normalisation) function in FSL (FMRIB Software Library) to obtain a measurement of the percentage of brain volume change between baseline and follow-up [29]. This process was entirely automated except for the choice of brain extraction parameters; the best set of input parameters to optimise brain extraction were selected on a subject by subject basis for each scan by one of the authors (JYG). The advantage of SIENA is that it uses the perpendicular distance along the image gradient direction between the brain edge at two time points, to estimate the shift of the brain edge and so estimate brain atrophy over the time. Thus, this method does not require intensity normalization of the original input images from two time points, which means it is less sensitive to problems arising from the difference pattern of image intensity histogram between two images [30]. As such, SIENA solves one of the common problems in all longitudinal studies, namely intensity differences in the images during the follow-up period due to hardware upgrades. Empirical research has shown SIENA to produce highly accurate results (i.e. close to the gold standard of manual segmentation) in evaluating brain volume change over time [31].

Regional SIENA statistics were obtained for the lateral edges of the frontal, temporal, parietal, and occipital lobes and cerebellum, as well as for peri-ventricular regions. This was achieved by dilating the edge displacement image for each subject, transforming it into MNI152 space, and masking it by a standard MNI152-space brain edge image. In this way, voxelwise edge displacement values were warped onto the standard brain edge. The average brain edge displacement value in mm was calculated for our regions of interest using a combination of the standard space brain edge image, and the MNI atlas supplied with FSL.

### The annual brain volume change

The annual brain volume change was calculated using the individual brain volume change percentage obtained from FSL-SIENA outputs and the exact length of follow-up of each individual.

### Cognitive function

We assessed change in a global measure of cognition at both times points (and hence global cognitive change) as follows. Cognition was assessed at both times with tests of executive function, working memory, visual and verbal learning, using the Abstraction, Inhibition and Working Memory test [32] (AIM), Visual Object Learning Test [33] (VOLT), and Californian Verbal Learning Test [34] (CVLT). From these tests, we obtained the following variables: total score of the first five learning trials from the CVLT, total score of the four learning trials from the VOLT, the abstraction score from the AIM and the abstraction and working memory score from the AIM. We calculated standardized scores for each cognitive variable using the mean and standard deviation of the cohort general population controls performance at baseline for standardisation purposes; this resulted in a control sample mean of 100 and standard deviation of 15 for each test. We then averaged these standardised scores across cognitive domains at baseline and again for follow-up in order to express a score representing global cognitive function for each assessment. Five schizophrenia participants and nine controls had missing data in more than one test and were excluded from the cognitive analysis.

### Statistical analysis

Statistical differences in mean brain volume change between the two groups were tested using an independent sample t-test and in a univariate general linear model, with group as a predictor and covariates of sex, educational level, alcohol use, and weight change.

Associations between brain volume change and independent clinical variables (total PANSS score, change in total PANSS score, PANSS sub-scores, changes in PANSS sub-scores, SOFAS score, change in SOFAS scores, change in cognition, and antipsychotic dose years) were assessed using linear regression. In the analyses of antipsychotic medication we used five different multivariate models including the following covariates: Model 1: alcohol use, weight change; 2: mean PANSS, alcohol use, weight change; 3: change in PANSS, alcohol use, weight change; 4: mean SOFAS, alcohol use, weight change; 5: change in SOFAS, alcohol use, weight change. Alpha was set to 0.05. PASW 18.0 statistical software was used for all the analyses. We report standardised regression beta parameters.

### Attrition analyses

The data collection is shown in Figure 1. Within the schizophrenia group, of the 101 invited participants 68 (67%) did not participate at both time points, baseline and follow-up. Of the non-participants 65% were males and 35% females, while of the 33 participants 58% were males and 42% females (p = 0.52, Fisher's Exact Test). In non-participants, 26% had 9 years or less basic education and 9% 12 years or more education; from the participants 21% had 9 years or less education and none had 12 years or more education (p = 0.17). The mean age of onset was 23.0 years in non-participants and 22.5 (p = 0.62, independent samples t-test) years in participants.

In the control group we evaluated how the 71 participants differed from the rest of eligible non-psychotic population of the NFBC 1966 (N = 10,490). The educational level of non-participants was lower than in participants: of the non-participants 15% had 9 years or less basic education, and 26% had 12 years or more education; of the participants 4% had 9 years or less education and 32% had 12 years or more education (p = 0.016).

## Results

**Figure 2:**
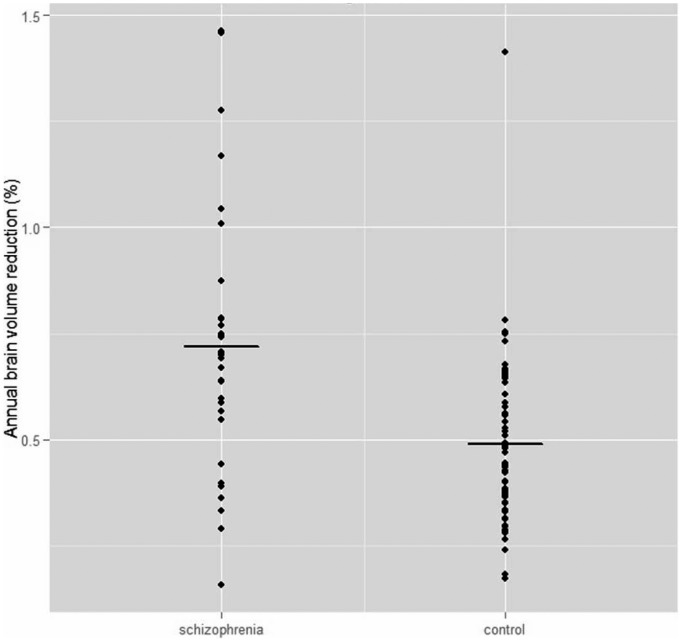
Scatter blot of annual brain volume change in schizophrenia and control groups.

The mean annual total brain volume reduction was 0.69% in participants with schizophrenia and 0.49% in control participants (Figure 2). The reduction in the schizophrenia group was statistically significantly higher than in the control group after adjusting for sex, educational level, alcohol use and weight change (Table 3). In seven participants (21%) with schizophrenia the annual total brain volume reduction was lower than the mean of total brain volume reduction in control participants.

**Table 3:** Mean of the annual change of total brain volume (%) during the 9-year follow-up period in participants with schizophrenia and control participants.

		Annual change of brain volume (%)					
	n	mean (SD)	Range	Q_1_, Q_3_	P-value^a^	P-value^b^	P-value^c^
					<0.001	<0.001	0.003
**Schizophrenia**	33	−0.69 (0.28)	−1.46, −0.16	−0.78, −0.57			
**Controls**	71	−0.49 (0.19)	−1.41, −0.17	−0.63, −0.36			

SD  =  Standard Deviation.

Q_1_  =  first quartile, Q_3_  =  third quartile.

a Independent samples t-test.

b Adjusted for sex.

c Adjusted for sex, educational level, logarithm of the alcohol use (g/day) and weight change (kg).

In linear regression analyses, total brain volume reduction did not associate with the mean total PANSS score, change in the total PANSS score, mean SOFAS score or change in SOFAS score in schizophrenia (Table 4). The brain volume reduction was not associated with the means of PANSS sub-scores or changes in PANSS sub-scores. Weight change (beta = −0.23, t = −1.3, p = 0.20) and alcohol use (beta = −0.39, t = 0.22, p = 0.83) did not predict brain volume change in schizophrenia.

**Table 4:** Linear regression between annual brain change and mean PANSS scores, change in PANSS scores, mean SOFAS and change in SOFAS score.

	beta^a^	t	p
**Mean Total PANSS**	**−0.09**	**−0.50**	**0.62**
Mean negative symptom subscore	0.10	0.48	0.64
Mean positive symptom subscore	−0.28	−1.42	0.17
Mean cognitive symptom subscore	−0.22	−1.11	0.28
Mean excitement symptom subscore	−0.14	−0.69	0.50
Mean depression symptom subscore	0.01	0.03	0.98
**Change in total PANSS**	−**0.19**	−**1.03**	**0.31**
Change in negative symptom subscore	−0.24	−1.20	0.24
Change in positive symptom subscore	−0.23	−1.17	0.26
Change in cognitive symptom subscore	0.01	0.03	0.98
Change in excitement symptom subscore	−0.38	−2.00	0.06
Change in depression symptom subscore	−0.05	−0.23	0.82
**Mean SOFAS**	**0.24**	**1.37**	**0.18**
**Change in SOFAS**	**0.13**	**0.72**	**0.48**

a Standardized betas.

The schizophrenia group (mean 79.3, SD 18.9) had a significantly lower global cognition score compared to controls (mean 100.0, SD 9.3) at baseline (independent samples test p<0.0001) and at follow-up (respective figures, 75.4 (SD 21.2) and 99.1 (SD 9.0); independent samples t-test p<0.0001). Global cognitive performance was stable: there was no significant change in either within group or between groups (p>0.1). Reduction of total brain volume in participants with schizophrenia was not significantly associated with decrease in overall cognition (r = 0.21, p = 0.28), or with decrease in specific cognitive processes (verbal learning r = 0.23, p = 0.25; visual learning r = 0.20, p = 0.33; abstraction r = −0.14, p = 0.46, abstraction with working memory r = 0.10, p = 0.61).

**Figure 3:**
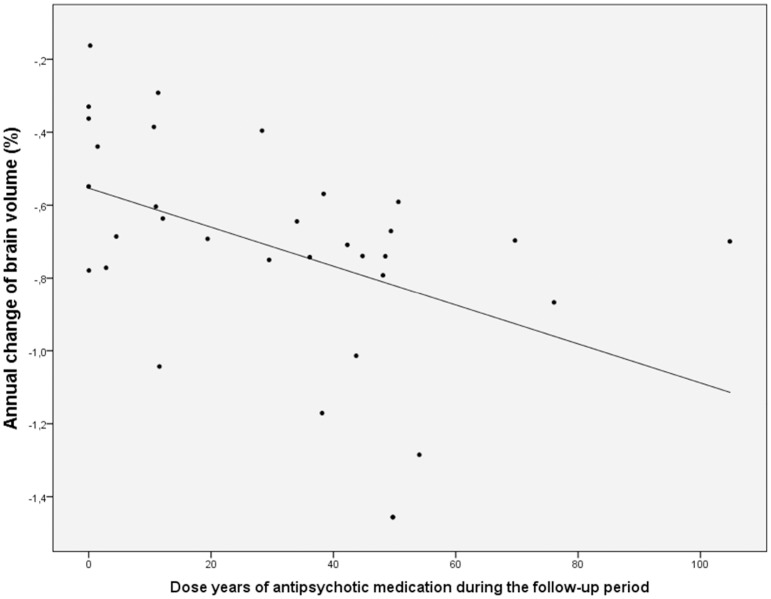
Linear regression between dose years (equivalent to daily 100 mg chlorpromazine) of antipsychotic medication and annual change in brain volume (%) in participants with schizophrenia (beta = −0.50, t = −3.23, p = 0.003). 1000 mg chlorpromazine daily for a duration of 9 years equals 90 dose years.

**Figure 4:**
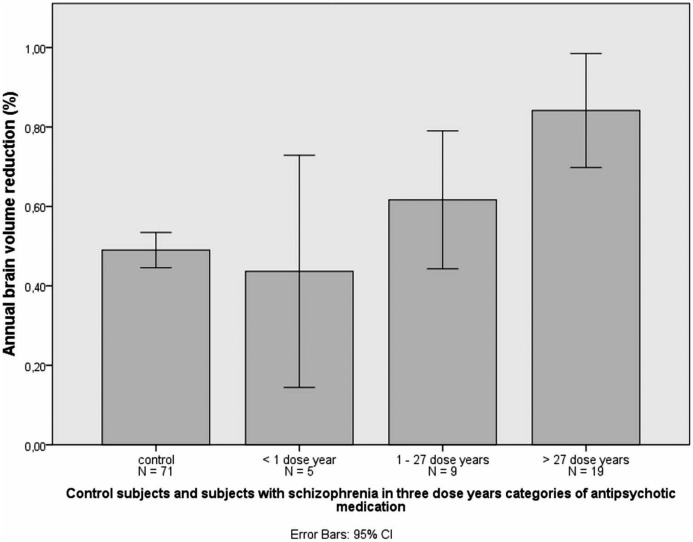
Annual brain volume reduction (%) in control participants and in participants with schizophrenia in three dose year (equivalent to 100 mg chlorpromazine daily) categories of antipsychotic medication. 300 mg chlorpromazine daily for a duration of 9 years equals 27 dose years.

Cumulative exposure to antipsychotic medication predicted reduction in brain volume in schizophrenia (linear regression; beta = −0.50, t = −3.23, p = 0.003, Figure 3). This effect remained significant (p<0.05) whether adjusting for symptom severity (mean total PANSS score or change in total PANSS score), social functioning (mean SOFAS score or change in SOFAS score), alcohol use, or weight gain (Table 5). Mean brain volume decrease in those five schizophrenia participants with less than 1 dose year of antipsychotic medication did not differ from control participants, but in those schizophrenia participants with more dose years, brain volume reduction was increased (Figure 4).

**Table 5:** Linear regression analysis of the association between annual brain volume reduction and amount of antipsychotic medication (log dose years of daily equivalent of 100 mg chlorpromazine separately in typical, atypical and any antipsychotics) during the 9-year follow-up time.

Medication	beta a	t a	p a	beta^b^	t^b^	p^b^	beta^c^	t^c^	p^c^	beta^d^	t^d^	p^d^	beta^e^	t^e^	p^e^
**Typical antipsychotics**	−0.30	−1.74	0.092	−0.29	−1.64	0.113	−0.35	−1.98	0.058	−0.22	−1.23	0.215	−0.31	−1.78	0.085
**Atypical antipsychotics**	−0.54	−3.53	0.001	−0.55	−2.95	0.006	−0.50	−2.98	0.006	−0.45	−2.27	0.031	−0.50	−3.15	0.004
**Any antipsychotics**	−0.50	−3.23	0.003	−0.53	−2.80	0.009	−0.48	−2.89	0.008	−0.42	−2.10	0.047	−0.48	−3.02	0.005

a unadjusted model. All betas are standardised.

b adjusted for mean total PANSS, logarithm of alcohol use and weight change.

c adjusted for change in total PANSS, logarithm of alcohol use and weight change.

d adjusted for mean SOFAS, logarithm of alcohol use and weight change.

e adjusted for change in SOFAS, logarithm of alcohol use and weight change.

In the sub-group of seven participants with schizophrenia in whom the annual total brain volume reduction was lower than the mean of total brain volume reduction in control participants, the mean dose years was 7.4 compared to 36.1 dose years (t = 2.9.0; df = 31; p = 0.007) in the rest of the schizophrenia group. When we compared the characteristics of patients who show more than control average brain volume change to patients who show less than control average brain volume change, we found no differences in gender, age of illness onset, or baseline marital status, symptoms severity (PANSS), social functioning, weight, alcohol use, or level of education. In separate linear regression analyses on the effects of typical and atypical antipsychotic medication, the annual reduction in brain volume was predicted by exposure to both types of antipsychotic medication with a high level of significance for atypical antipsychotic medication (atypical beta = −0.54, t = −3.53, p = 0.001) and a marginal significance for typical antipsychotic medication (typical beta = −0.298, t = −1.74, p = 0.092). After adjusting for symptom severity (mean or change in total PANSS score, or change in SOFAS score), alcohol use and weight gain, the associations remained statistically significant for atypical medication (Table 5).

Regional measures of brain change are presented in Table 6: group differences in brain reduction were most prominent in the temporal lobe and periventricular area. In regional analysis of the association between dose years of antipsychotic medication and brain reduction, there were associations in the parietal lobe (spearman's rho = 0.38, p = 0.03), temporal lobe (rho = 0.45, p = 0.008) and the periventricular area (spearman's rho = 0.55, p = 0.001). In these areas, greater doses of medication were associated with more pronounced brain reductions; however, there were no significant associations in the frontal lobe (rho = 0.28, p = 0.12), occipital lobe (rho = 0.21, p = 0.25) or cerebellum (rho = 0.14, p = 0.42).

**Table 6:** Mean brain edge movement (reduction) by brain region in schizophrenia and during the 9-year follow-up time.

	Mean edge movement in mm (SD)		
	Schizophrenia	Controls	P*
Frontal lobe	−0.31 (0.21)	−0.24 (0.13)	0.07
Parietal lobe	−0.21 (0.21)	−0.15 (0.13)	0.053
Temporal lobe	−0.22 (0.17)	−0.14 (0.07)	0.001
Occipital lobe	−0.35 (0.29)	−0.30 (0.24)	0.34
Cerebellum	−0.33 (0.19)	−0.34 (0.15)	0.75
Lateral ventricles	−0.35 (0.43)	−0.21 (0.16)	0.02

Note: *p-values from group comparison adjusted for sex.

## Discussion

Two out of our three hypotheses gained support in the study. We found greater loss of brain volume in participants with schizophrenia than in control participants during the follow-up period. Use of antipsychotic medications was associated with excessive brain loss. Our third hypothesis, that in people with schizophrenia the decrease in total brain volume would be associated with symptom severity, level of function, and deterioration in cognition, was not supported.

In this birth cohort based study, we found greater loss of brain volume in participants with schizophrenia compared to control participants drawn from the general population between the ages of 34 and 43 years over a nine year follow-up period. In other words, we demonstrate progressive brain volume loss in participants with schizophrenia in early middle age. Meta-analyses of previous studies comparing progressive volume changes in ward-based or clinic selected schizophrenic patients and controls recruited from the community by advertisement have also demonstrated progressive loss of volume in schizophrenia, notwithstanding the significant heterogeneity in the results as well as negative findings [2], [3]. Our general population based recruitment strategy complements previous reports based on clinical samples, and our results confirm that previously observed case-control differences are not secondary to recruitment bias, which has previously been shown to be an influential factor in schizophrenia brain volumetric imaging studies [10]–[12].

Although the schizophrenia group as a whole did exhibit a greater degree of reduction in brain volume than the control group, we observed considerable within group variability of brain volume changes and notable overlap between groups: 21% (7 out of 33 participants) of schizophrenia participants lost no more brain volume over time than the control average.

We investigated a clinically important potential determinant of the degree of brain volume changes in schizophrenia: the effect of antipsychotic medication. We have shown here that there was a predictive relationship in schizophrenia between exposure to antipsychotic medication and brain volume loss over time. Observational studies may be susceptible to confounds however, and in interpreting these findings it is necessary to consider the effect of confounding or moderating factors, such as illness severity. It is conceivable that patients with the most severe illness lose more brain volume over time, reflecting intrinsic aspects of the pathology of schizophrenia, and the fact that severely ill patients receive higher doses of medication. Crucially, the association between brain volume change and medication dose remained statistically significant even after controlling for illness severity. The association remained robust after controlling for a variety of other potential confounding factors, including weight change and alcohol consumption. One of the strengths of this study stems from the observation that recreational drug use was rare in our sample, and thus unlikely to be an important contributor to the results.

Our finding that antipsychotic medication is related to brain volume loss in a dose-dependent manner is consistent with a previous report from the Iowa Longitudinal Study, which found similar results in a large clinic-based sample of young adults followed over an average seven year period [6]. Additionally, we found some evidence that atypical antipsychotic medication may have an effect on brain volume loss. These results are in contrast to a study from the Netherlands, which found that only typical, but not atypical, antipsychotic medication predicted cortical thinning in schizophrenia [7].

Both typical and atypical antipsychotic agents have been shown to produce loss of brain volume in experimental rodents [35] and monkeys [36], suggesting that antipsychotic drugs have the potential to cause loss of brain volume over time. However, the mechanism through which this might occur is unclear, and it is conceivable that the differences between individual drugs may reflect the degree to which they may exert such effects [37]. In cell culture studies, a number of antipsychotics have been shown to induce autophagy, a process involved in neurodegenerative processes and cell death [38], suggesting a possible mechanism by which antipsychotics might cause loss of brain volume.

Antipsychotic medications are effective in treating psychotic symptoms in schizophrenia, and in preventing relapse [39], [40]. It is equally clear that they also have the potential to cause serious adverse effects for example tardive dyskinesia. Indeed, over thirty-five years ago, Marsden hypothesized that “long-term neuroleptic therapy could explain some of the cerebral atrophy” in schizophrenia, the first demonstrations of which were emerging at the time [41], [42]. Careful deliberation over risks and benefits is shared between clinicians and patients when it comes to decisions about medication strategies.

It is possible that antipsychotic medications may have differential effects on brain volume in different psychiatric and neurological disorders, or at different stages of the lifespan or at different stages of illness. Antipsychotic medications are used increasingly, whether on or off-label, in treating a variety of psychiatric conditions, including dementia, bipolar disorder, unipolar depression, and in a variety of child psychiatric conditions; to date no studies have been published examining their long-term effects on brain volume in conditions other than schizophrenia.

The regional findings in our study suggest that the excessive atrophy in patients compared to controls predominantly affects periventricular regions and the temporal lobe. There were differences between groups in volume change in the parietal and frontal lobe, but these were not statistically significant, whereas we found no evidence of differential atrophy in schizophrenia in the occipital lobe and cerebellum. We found that the association between exposure to antipsychotic medication and atrophy was prominent in the periventricular region, which is consistent with results reported by Ho and colleagues, although we could not replicate their findings of cerebellar loss associating with cumulative medication [6].

Whilst it is extremely important to determine the causes of loss of brain volume in schizophrenia, an equally important question concerns its clinical significance. Loss of brain volume occurs throughout the majority of adult life in the healthy population, and whilst it might seem trivial that this would be disadvantageous, in some periods of development loss of brain tissue appears to be potentially beneficial [43]. We examined three different outcome variables of interest: level of symptom, level of functioning, and cognition. We found no evidence that loss of brain volume had disadvantageous effects on global levels of symptomatology, global cognition or level of function, though we recognize that this does not preclude the possibility that loss of brain volume may have important functional consequences (perhaps on specific cognitive domains or specific symptoms) that our study did not identify. Future studies should address this important question using sensitive neuropsychological instruments.

### Strengths and limitations

Our study has several strengths. We studied participants with schizophrenia who were drawn from the general population, rather than a clinical setting. The control participants of the present study were matched for age (birth year) and birth place, and were randomly selected from the general population rather than being recruited by advertisements. Thus, we can be confident that the differences in progressive loss of brain volume that we observed in participants with schizophrenia compared to control participants were not secondary to recruitment biases. Our results show that loss of brain volume in schizophrenia is not confined to the early stages of the illness. We believe that the measurements for dose years of antipsychotic medication during the follow up were reliable as we used multiple sources of information. We acknowledge that prescribed doses are not always equal to consumed doses.

The relatively modest number of participants with schizophrenia is the main limitation of the study, and this limited our ability to examine whether different medications had differential effects on brain volume change. Nevertheless, our sample size is one of the largest and the follow-up one of the longest in the field. Furthermore our attrition analyses showed that subject drop-out is unlikely to have affected the results. Although our control participants are more educated than the patients, and than the average Finnish person born in Northern Finland in 1966, our control group had fewer years of maternal education in comparison to the maternal education of our patient group. This means that the group differences we observed in progressive brain volume loss are unlikely to be solely explicable in terms of social and education selection biases.

We were unable to assess the role of some potentially relevant factors that could putatively drive brain volume changes over time: for example we did not have sufficient data to examine the role of physical exercise in predicting brain volume changes, although we did not find evidence that weight gain predicted global brain volume loss.

Although our results show that atypical (specifically) antipsychotic medication use may predict the degree of loss of brain volume, we note that a previous randomized controlled trial provided evidence that different antipsychotic medications may have differential effects on brain volume [44]. Another limitation is that although we used the same MRI scanner bore at both time points, much of its hardware was updated during the period between the initial scan and the follow-up scan, most notably by using different gradients and a different multi-channel data acquisition head coil at follow-up. Scanner upgrades are a notable problem in most long-term neuroimaging studies. However, we used an analysis method that has been shown to be extremely robust to changes in data acquisition protocols over time to circumvent this limitation; the method of image analysis that we used, SIENA, has been shown to provide superior performance in the analysis of brain volume change over time in comparison to other commonly used image analysis techniques [31].

## Conclusions

Continuing brain volume reduction occurs in schizophrenia in early middle-age. However, there is considerable inter-individual variability in the rate of brain volume reduction. At present, the clinical significance of brain volume reduction is uncertain and further research on this topic is of critical importance [45]. Antipsychotic medications may contribute to progressive brain volume reduction in schizophrenia, in accordance with previous predictions based on clinical and preclinical research [8], [35], [36], [40].
